# Time Pressure and Health-Related Loss of Productivity in University Students: The Mediating Role of Exhaustion

**DOI:** 10.3389/fpubh.2021.653440

**Published:** 2021-04-27

**Authors:** Burkhard Gusy, Tino Lesener, Christine Wolter

**Affiliations:** Department of Education and Psychology, Division of Public Health: Prevention and Psychosocial Health Research, Freie Universität Berlin, Berlin, Germany

**Keywords:** study demands-resources framework, time pressure, student burnout, health-related loss of productivity, student well-being

## Abstract

**Introduction:** Being present at work when sick is not just prevalent in employees. Since university is also a demanding context, there is a growing interest in this phenomenon in university students. Especially students with mental health issues show a higher degree of productivity loss. However, little research has examined the causes of these productivity losses—especially in university students. Therefore, we examined health-related (burnout) and non-health-related (time pressure) aspects that lead to productivity losses in the long run.

**Methods:** We decided to examine the effect from time pressure on health-related loss of productivity, mediated by exhaustion. This assumption is in line with the health impairment process proposed by the Study Demands-Resources (SD-R) framework. To examine this assumption properly, we conducted a longitudinal study with three occasions. We surveyed 392 students in three waves over 1 year and performed structural equation modeling (SEM) to confirm the assumptions longitudinally.

**Results:** In line with our assumptions, time pressure predicted burnout which, in turn, predicted health-related loss of productivity in the long run. Hence—as assumed by the SD-R framework—burnout serves as a mediator between study demands and negative outcomes such as loss of productivity.

**Discussion:** Our study is the first that uncovers health-related and non-health-related causes of health-related productivity loss in university students. Thus, we were able to confirm SD-R's health impairment process longitudinally. Since we know that time pressure serves as a major antecedent for burnout and health-related loss of productivity, we are well-advised to establish appropriate interventions to reduce students' time pressure.

## Introduction

Many people know the feeling of having to go to work even when too sick or stressed to be productive (1). In these moments, they may experience decreased productivity and below-normal work quality (2). This concept is well-known as presenteeism—or a health-related loss of productivity (2). Health-related loss of productivity is a widespread and costly issue: 39% of the EU workforce work despite being ill (3) and 70% of German employees report having been sick at work on at least 1 day within the previous year (4). However, this phenomenon cannot only be found in workers: there is also growing interest in the health-related loss of productivity in university students (5). Especially students with mental health issues show a higher degree of productivity loss than those with other issues [e.g., physical issues; (5)].

However, there is still little research on the causes of health-related loss of productivity—especially in university students. Therefore, our aim is to investigate the longitudinal relationships between study demands, student burnout, and the health-related loss of productivity among students of a large German university.

In the occupational context, several reasons have been identified for why employees go to work when they are sick, including perceived pressure from colleagues, the worry about career opportunities, or even the fear of termination (1). Most empirical research examining the antecedents of health-related loss of productivity has focused on health-related issues, such as specific conditions (e.g., stress) or overall indicators of self-rated health (6). These studies suggest that poor health is a key indicator for productivity losses in the workplace (7). However, there are also non-health-related issues that have been associated with health-related loss of productivity (6), including the relationship with colleagues (8), job insecurity (9, 10), high workload (1, 11), or time pressure (8). While some of these issues are only relevant in the occupational context, others also apply to the university context. We decided to focus on the relationship between one non-health-related issue (time pressure) and one health-related issue (student burnout), and the outcome of health-related productivity loss.

Time pressure can be understood as an increase in workload resulting in a lack of time and often in a decrease of leisure time (12). It has already been identified as one of the key stressors at university (13–15), which is related to stress and depressive symptoms. For almost two thirds of the students, time pressure is the key stressor of university life (13). Several studies have shown strong relationships of time pressure with student burnout (16, 17). As mentioned above, time pressure has been identified as an antecedent of health-related loss of productivity among employees. However, empirical research on this relationship among university students is missing.

**Figure 1:**
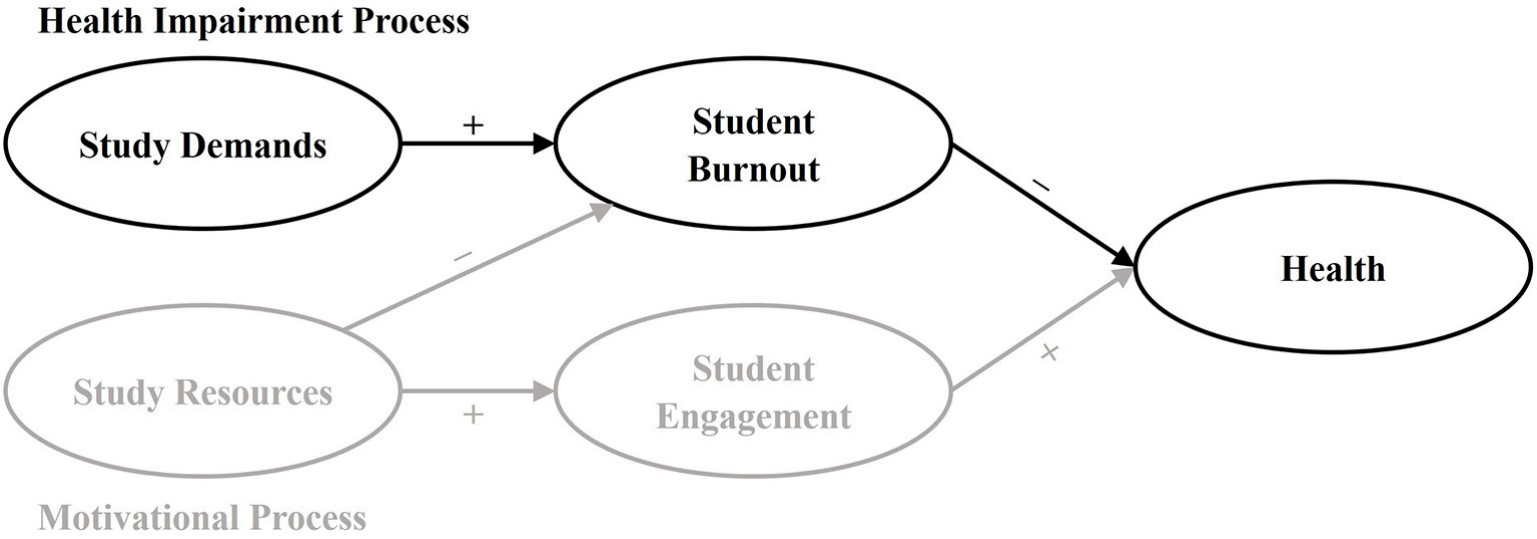
The study demands-resources (SD-R) framework.

Student burnout is also an important issue regarding students' health and well-being. Especially exhaustion—the initial symptom of burnout, which shows the stressors' effect on the individual stressor—is common among students even when compared to employees that report high rates of exhaustion such as physicians (18). Almost 25% of university students suffer from severe symptoms of exhaustion (19, 20). Burnout is related to impaired health and well-being (21–23), at least cross-sectionally. In the occupational context, exhaustion has been identified as an antecedent of health-related productivity loss (1, 24). However, empirical results on the effect within the university context are missing (18). To clarify the relationship between health-related loss of productivity, exhaustion, and time pressure, we used the Study Demands-Resources [SD-R; (17)] framework (see Figure 1). The SD-R framework is an influential theoretical basis to examine salutogenic and pathogenic effects of the study context on students' health and well-being. It is an application of the well-established Job Demands-Resources (25–27) framework to the university context. The framework proposes that poor study program design and excessive study demands lead to student burnout and health problems in the long run, whereas study resources lead to higher student engagement and better performance (17). Study demands are those physical, social, or organizational aspects of studying that require sustained physical or mental effort, and are therefore associated with certain physiological and psychological costs (17, 26). Student burnout is defined as a consequence of extended exposure to specific study demands like intense physical, affective, and cognitive strain (20, 28). The final outcomes of the SD-R framework are various positive or negative health- and performance-related indicators such as life satisfaction, academic performance, health complaints, dropout—or a loss of productivity.

The SD-R framework implies that high study demands increase the risk for student burnout and lead to negative outcomes, such as the health-related productivity loss (17). Lesener et al. (17) were able to validate these essential assumptions cross-sectionally. The framework has also been applied and validated in various occupational and organizational contexts—longitudinally and even meta-analytically. However, within the study context, SD-R's essential assumptions have not yet been tested longitudinally. To examine these assumptions properly, we need studies with at least three occasions.

To our knowledge, our study is the first that examines the longitudinal relationship between time pressure as the major study demand, exhaustion, and the health-related loss of productivity. In line with SD-R's health impairment process—we hypothesize:

Hypothesis 1: Time pressure leads to student burnout over time.
Hypothesis 2: Student burnout leads to health-related loss of productivity over time.
Hypothesis 3: Student burnout mediates the longitudinal effect from time pressure on health-related loss of productivity.

## Materials and Methods

### Study Procedure and Sample

Our study was part of a regular health monitoring survey at a major university in Germany. We invited all students to take part in the study. We surveyed the students on three occasions, each 6 months apart. The time lag of 6 months between each occasion is very common for three wave studies to identify antecedents and outcomes of burnout (27). Our aim was to survey students at the end of the semester before the exam period had started.

We invited 33,267 students to take part in our study. Three thousand four hundred twenty students completed the questionnaire at T1, and 1,245 provided their e-mail address to take part at T2 (*n* = 866) and T3. In total, 392 students completed the questionnaire on all three occasions, resulting in a response rate of 10% at T1, 25,2% from T1 to T2 and 45,3% from T2 to T3. Our final sample consisted of 290 women (74%) and 95 men from all departments of this university (Biology, Chemistry, and Pharmacy, Earth Sciences, Education and Psychology, History and Cultural Studies, Law, Mathematics and Computer Science, Philosophy and Humanities, Physics, Political and Social Sciences, Veterinary Medicine, Business and Economics). The mean age of our respondents was 24.4. years (*SD* = 5.5 years) and they were, on average, in their third year of studying (range = 1–9 years). Differences between longitudinal and cross-sectional participants were examined using t and Chi-Square tests. There were no significant differences between the two groups in either sociodemographic characteristics (age, gender, duration of study, intended degree) or analysis characteristics (time pressure, exhaustion, health-related productivity loss. Table 1).

**Table 1:** Means, standard deviations, and differences in age, gender, duration of study, time pressure, exhaustion and health-related loss of productivity between cross-sectional and longitudinal participants at T1.

		**T1 (*****N*** **=** **3.025)**		**T1-T2-T3 (*****N*** **=** **392)**				**95% CI**	
		**M**	**SD**	**M**	**SD**	**T**	***P***	**LL**	**UL**
1	Age	24.06	5.52	14.07	5.5.2	0.077	0.94	−0.52	0.55
2	Gender	Women (*N* = 1.819)	Men (*N* = 699)	Women (*N* = 290)	Men (*N* = 95)				
3	Duration of study	7.09	4.80	7.04	4.98	−0.20	0.84	−0.58	0.47
4	TP	3.33	1.04	3.39	1.04	1.25	0.21	−0.04	0.18
5	EX	2.75	1.63	2.68	1.54	−0.78	0.43	−0.24	0.10
6	HLP	2.22	1,22	2.17	1.19	−0.71	0.48	−0.17	0.08

*TP, time pressure; EX, exhaustion (MBI); HLP, health-related loss of productivity; T1, time 1; T2, time 2; T3, time 3*.

### Measures

#### Time Pressure

To capture time pressure as the major study demand, we used a self-constructed scale that has been successfully applied in various health monitoring surveys at universities [e.g., (19, 29)]. The three items included in the survey identify study demands induced by a subjective scarcity of time (see Supplementary Material). All items were answered using a Likert scale ranging from “never” (1) to “always” (6). The internal consistency in our study was α = 0.80 (T1).

#### Exhaustion

To assess student burnout, we used the exhaustion sub-scale of the Maslach Burnout Inventory–Student Form [MBI-9-SF; (20, 30)]. The sub-scale consists of three items (see Supplementary Material). The frequency of these experiences is scored from “never” (0) to “daily” (6). The sub-scale's mean score is computed, and high scores are indicative of higher student burnout. The factorial validity of the abbreviated MBI-SF scales has been confirmed (20), and the internal consistency of the sub-scale in our study was α = 0.83 (T1).

#### Health-related Loss of Productivity

To capture the health-related loss of productivity, we applied the Stanford Presenteeism Scale (SPS) developed by Koopmann et al. (2). This instrument, adapted for students, measures the health-related loss of productivity within the university setting (2). We used five items of the SPS (see Supplementary Material). All items were answered using a Likert scale ranging from “does not apply at all” (5) to “applies completely” (1). The internal consistency of this scale in our study was α = 0.94 (T1).

### Data Analysis

To test our hypotheses, we performed structural equation modeling (SEM) using Mplus version 8.4. As recommended by Hu and Bentler (31), we assessed the models' goodness of fit by Tucker–Lewis Index (TLI), and Comparative Fit Index (CFI), root mean square error of approximation (RMSEA), standardized root mean square residual (SRMR). TLI and CFI are less sensitive to the number of observations. An RMSEA value of <0.06 and a SRMR value of 0.08 or lower indicate good model fit (32). For TLI and CFI, values of 0.90 may be interpreted as an acceptable fit (33).

**Figure 2:**
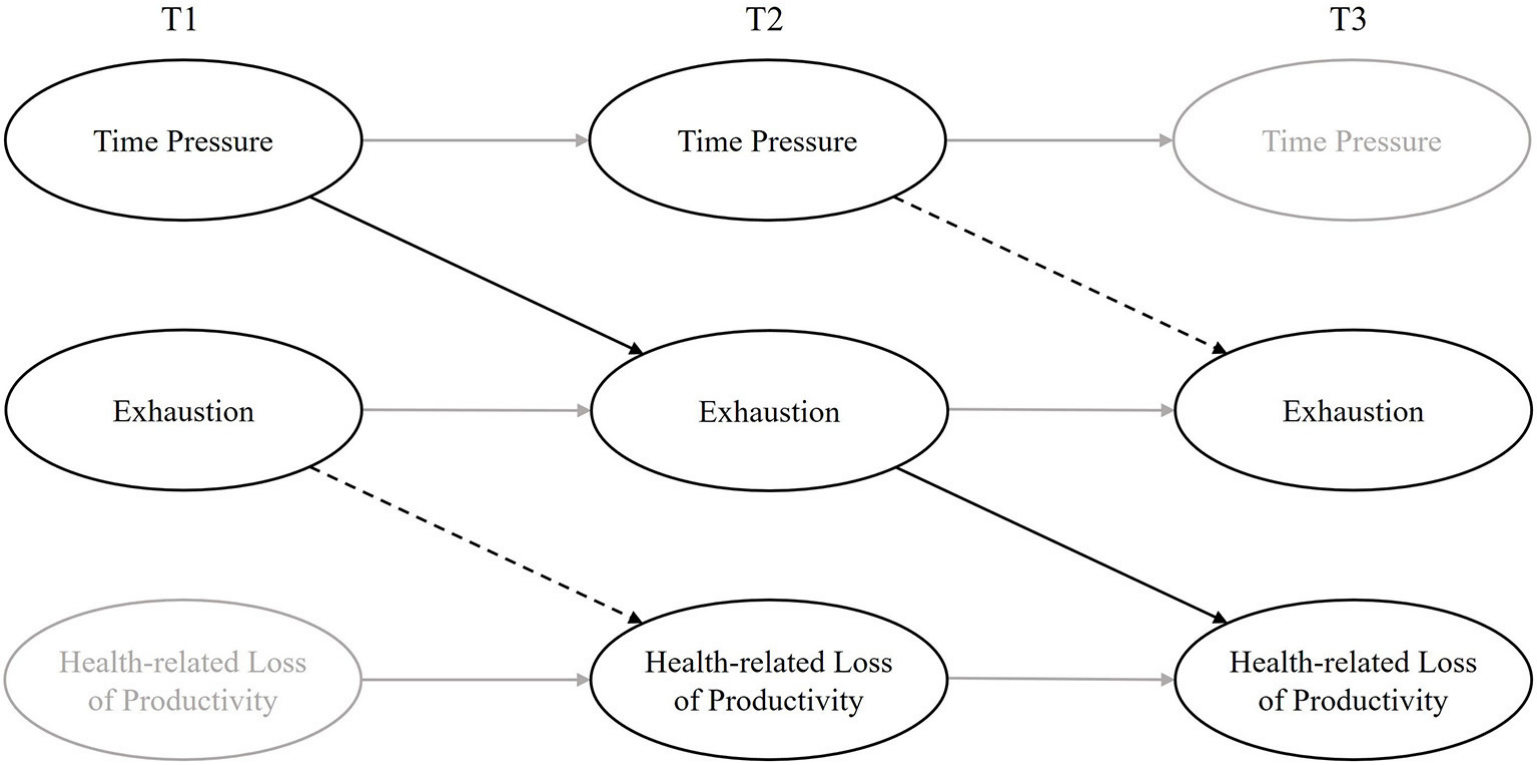
The hypothesized model. All exogenous latent constructs are represented by manifest variables shown in Table 2.

To test the longitudinal effect of time pressure on health-related loss of productivity mediated by exhaustion, we used the data of a three-wave survey. We examined the temporal relationships between time pressure, exhaustion, and health-related loss of productivity using cross-lagged panel models (CLPM). CLPM are most popular for examining temporal associations between three variables (34), since they control for several biases (i.e., the stability of the variables, cross-sectional associations, and prior associations between the variables). To test mediation models properly, CLPM with three occasions are favorable (34). Therefore, we followed the guidelines for mediation models for longitudinal data made by Preacher (34) (see Figure 2).

First, we specified a model (M0), which only included the autoregressive effects of the three variables over time. In a second model (M1; see Figure 2) we added the paths of interest as follows: we included the cross-lagged paths from time pressure (T1) to exhaustion (T2) and from exhaustion (T2) to health-related loss of productivity (T3). The causality would be additionally supported if the time-lagged paths from exhaustion (T1) to health-related loss of productivity (T2), and from time pressure (T1) to exhaustion (T2) would be significant (see Figure 2: dotted lines). Then we evaluated two alternative nested models, one model with reversed causal effects (M2, see Supplementary Material) and one model with reciprocal effects (M3, see Supplementary Material). We compared the nested models (M0-M3) using the Akaike Information Criteria (AIC). The proposed model including our hypotheses (M1) should have a lower value than the model including only autoregressive paths. Additionally, we used indirect effect size estimates to confirm whether exhaustion serves as a (complete or partial) mediator between time pressure and health-related loss of productivity. Therefore, we added a direct path from time pressure (T1) to health-related loss of productivity (T3). If this path becomes significant and the model fits the data better, we can assume a partial mediation (35). If the model fits the data worse, we can assume a full mediation.

**Table 2:** Means, standard deviations, correlations and Cronbachs Alpha of the latent variables.

		**M**	**SD**	**1**	**2**	**3**	**4**	**5**	**6**	**7**	**8**	**9**
1	TP T1	4.65	1.12	(0.83)								
2	TP T2	4.69	1.13	0.69	(0.87)							
3	TP T3	4.59	1.12	0.63	0.72	(0.88)						
4	EX T1	2.68	1.54	0.52	0.47	0.45	(0.80)					
5	EX T2	2.62	1.70	0.48	0.60	0.53	0.74	(0.84)				
6	EX T3	2.63	1.67	0.48	0.52	0.60	0.69	0.73	(0.94)			
7	HLP T1	2.12	1.19	0.36	0.33	0.30	0.50	0.46	0.45	(0.96)		
8	HLP T2	2.12	1.19	0.19	0.27	0.27	0.48	0.48	0.45	0.58	(0.96)	
9	HLP T3	2.20	1.27	0.22	0.20	0.30	0.40	0.39	0.48	0.49	0.51	(0.96)

*TP, Time Pressure; EX, exhaustion (MBI); HLP, Health-related Loss of Productivity; Cronbachs Alpha in parenthesis; T1, time 1; T2, time 2; T3, time 3*.

## Results

Means, standard deviations, and correlations of the study variables are reported in Table 2. The correlation matrix of the manifest variables used for the analyses can be found in the Supplementary Material.

### Measurement Model

We specified and tested the measurement model of all latent constructs shown in Figure 2 prior to model testing. All constructs were assessed by 3–5 items. The overall measurement model with all manifest variables (time pressure, exhaustion, and health-related loss of productivity) on all occasions showed an acceptable fit (χ^2^ (459) = 1,009.11, *p* < 0.01; RMSEA = 0.06; SRMR = 0.04; TLI = 0.94; CFI = 0.94. All items loaded solidly on their respective factors (0.71 ≤ β ≤ 0.92; *p* < 0.001). To test measurement invariance over time, we introduced measurement-time-specific factors for time pressure, exhaustion, and health-related loss of productivity across the three measurement points (36). The model fitted the data significantly better than the unrestricted model. The standardized loadings of the measurement-time-specific indicators were <0.40 and can be classified as low to moderate. We can therefore assume configural measurement invariance for all three study variables over time.

### Structural Equation Model

In a second step we tested the model which only includes the autoregressive effects of each latent variable over time (time pressure, exhaustion, and health-related loss of productivity; M0). This model also showed an acceptable fit (χ^2^ (459) = 1,194.954, *p* < 0.01; RMSEA = 0.06; SRMR = 0.08; TLI = 0.93; CFI = 0.93; AIC = 35,585.22). Exhaustion (0.84 ≤ β ≤ 0.91) and time pressure (β = 0.86) were more stable than health-related loss of productivity (0.56 ≤ β ≤ 0.64).

**Figure 3:**
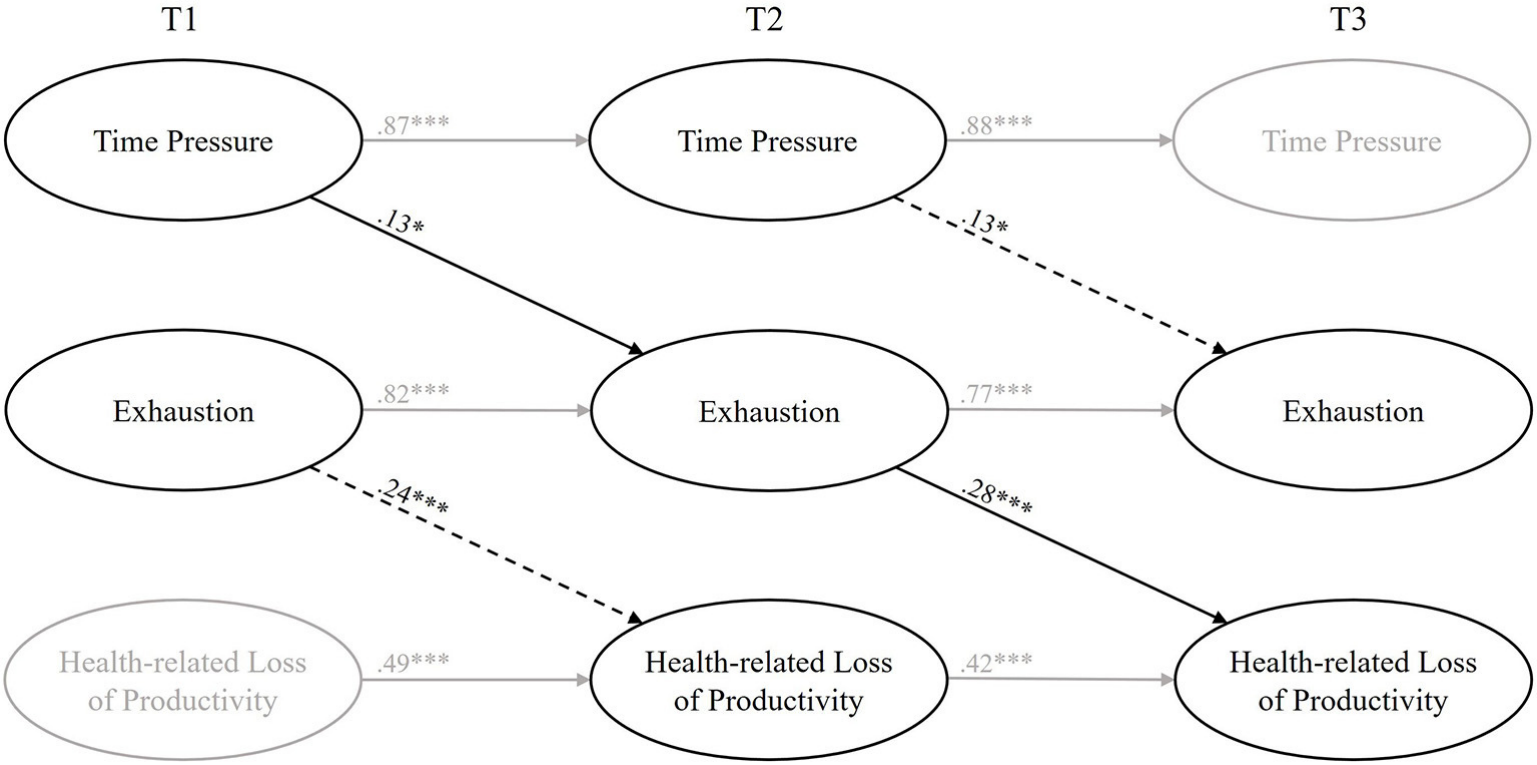
The final model. The manifest variables are not shown in this figure. *p > 0.05; **p > 0.01; ***p > 0.001.

Then we added the paths as mentioned above (M1; see Figure 2). The final model is depicted in Figure 3. The final model showed an acceptable fit (χ^2^ (459) = 1,141.36, *p* < 0.01; RMSEA = 0.06; SRMR = 0.06; TLI = 0.93; CFI = 0.93; AIC = 35,534.04). Comparing the AIC of the competing models (M0, M1), the final model (M1) showed a better fit. The autoregressive effects are slightly smaller compared to M0.

Hypothesis 1 postulated that time pressure leads to student burnout over time. Indeed, time pressure at T1 significantly predicted exhaustion at T2 (β = 0.13; *p* < 0.05). Furthermore, time pressure at T2 significantly predicted exhaustion at T3 (β = 0.13; *p* < 0.05). These results support Hypothesis 1.

Hypothesis 2 postulated that student burnout leads to health-related loss of productivity over time. As we can see, exhaustion at T1 significantly predicted health-related loss of productivity at T2 (β = 0.24; *p* < 0.001), and exhaustion at T2 significantly predicted health-related loss of productivity at T3 (β = 0.28; *p* < 0.001), which supports Hypothesis 2.

Hypothesis 3 postulated that student burnout (T2) mediates the longitudinal effect from time pressure (T1) on health-related loss of productivity (T3). To test this hypothesis, we added a direct path from time pressure (T1) to health-related loss of productivity (T3). Since this model fitted the data worse (χ^2^ (459) = 1,141.44, *p* < 0.01; RMSEA = 0.06; SRMR = 0.06; TLI = 0.93; CFI = 0.93) and the AIC increased (AIC = 35,535.70), we can assume that exhaustion fully mediates the effect from time pressure (T1) to health-related loss of productivity (T3). This approach is in line with the requirements for mediation models postulated by Dormann et al. (35). The bias-corrected bootstrap interval for the indirect effect from time pressure (T1) on health-related loss of productivity (T3) (CI 95% 0.01–0.12) indicates a significant indirect effect from time pressure (T1) to health-related loss of productivity (T3).

After testing M1, we tested the model with reversed causal effects (M2). The AIC for this model was worse compared with M1 (AIC = 35,576.13), the relevant longitudinal path from health-related loss of productivity (T1) on exhaustion (T2) was small and non-significant (β = −0.07; *p* > 0.05). Also, the longitudinal path from exhaustion (T2) on time pressure (T3) was small and non-significant (β = 0.07; *p* > 0.05). However, only the longitudinal effect from exhaustion (T1) to time pressure (T2) was significant (β = 0.13; *p* < 0.05) (see Supplementary Material).

Finally, we tested the model with reciprocal causal effects (M3). The AIC for this model was slightly better than for M1 (AIC = 35,529.475). However, only two longitudinal paths in this model were significant: the path from exhaustion (T1) on health-related loss of productivity (T2; β = 0.23; *p* < 0.001) and from exhaustion (T2) on health-related loss of productivity (T3; β = 0.27; *p* < 0.001) (see Supplementary Material). Only in M1 all longitudinal paths could be statistically validated. Since this was not the case for either the reversed model (M2) nor the reciprocal model (M3), we decided to retain M1.

## Discussion

Our study examined how time pressure, student burnout and health (health-related loss of productivity) are related over time. We adopted a three-wave panel design to establish a better understanding of the antecedents of health-related productivity loss. As hypothesized, time pressure leads to exhaustion, and exhaustion leads to health-related productivity loss over time. Hence—as assumed by the SD-R's health impairment process—burnout serves as a mediator between time pressure and the health-related loss of productivity.

In line with our predictions, time pressure showed to be positively related to a change in exhaustion, and exhaustion showed to be predictive for changes in health-related loss of productivity within a time-interval of 6 months. Adachi and Willoughby (37) claim that regression coefficients in longitudinal research are often much smaller than those in cross-sectional research. The authors argue that even smaller longitudinal regression coefficients are substantial, especially if the autoregressive effects are large (37). Furthermore, the longitudinal regression coefficients we found in our study are comparable to those reported in other longitudinal studies on student well-being [e.g., (38, 39)].

There is a controversial discussion about where to situate health-related loss of productivity within the SD-R framework. To our knowledge, two other studies have examined the relationship between (job) demands, burnout, and health-related productivity loss. In line with our perspective, McGregor et al. (6) considered health-related productivity loss as an outcome in the SD-R framework. However, due to the lack of longitudinal data, the authors were not able to test their assumptions properly. In contrast, Demerouti et al. (1) considered presenteeism to be a behavioral pattern that leads to burnout. In their three-wave study, they showed that (job) demands (T1-T2) significantly predicted exhaustion and presenteeism (only T2-T3) in the long run. Furthermore, exhaustion predicted presenteeism (T1-T2) and (job) demands (T1-T2), but they did not analyze any possible mediating effects of exhaustion (1).

When we only consider the lagged effect from time pressure on exhaustion, our results are in line with those of Demerouti et al. (1). As in their study, we have verified a lagged effect from time pressure on exhaustion. However, a lagged effect from exhaustion (T1-T2) on time pressure (T2-T3) is not consistent with our data. We could also show the lagged effect from exhaustion on health-related loss of productivity for both time intervals. This confirms the idea of loss spirals suggested by Hobfoll (40), in that exhaustion leads to reduced productivity, and underlines the necessity to recover after intense studying. However, reciprocal effects between both constructs are also conceivable since health-related loss of productivity in turn may increase exhaustion, although Demerouti et al. (1) found this effect for only one interval (T2-T3). Therefore, we tested the reciprocal relationships (M3) between health-related productivity loss (T1-T2) and exhaustion (T2-T3) and found that these paths were not significant for either interval.

## Strengths and Limitations

Our study is among the first that examines health-related (burnout) and non-health related (time pressure) causes of health-related loss of productivity as assumed by SD-R's health impairment process. Nevertheless, there are some issues we have to address below.

First, all variables were measured with self-reports, which might cause biases due to common method variance. However, we measured the variables with well-established and evaluated instruments. Demerouti et al. (1) operationalized their (job) demands more heterogeneously (workload, patient demands, physical demands), while McGregor et al. (6) used the Burgen Bullying Index. We focused solely on time pressure, a major predictor in burnout research. McGregor et al. (6) measured health-related loss of productivity with only one item (the total number of days lost at work due to presenteeism in the past year), whereas Demerouti et al. (1) directly asked whether participants had gone to work despite feeling sick in the past year. In contrast to them, we used the Stanford Presenteeism Scale, a well-established instrument that measures health-related loss of productivity with several items.

Second, even though CLPM is the most appropriate method for mediation analyses, it tends to overestimate the stability (autoregressive effects) of constructs and to underestimate the cross-lagged effects (41). Following this argument, the presented paths for example, from time pressure (T1) to exhaustion (T2) and from exhaustion (T2) to health-related loss of productivity (T3) might be larger than those shown in our analysis.

Third, lagged effects and fit indices of longitudinal models can change tremendously depending on the chosen time lag between the occasions. If the time lag between the occasions is too short, possible existing effects may not be detected. If the time lag between the occasions is too long, the effect may be underestimated (42). In studies regarding the consequences of health-related sickness presenteeism on health and well-being, the time lag was 2 to 12 months (43). As we know from our analysis, the stability of time pressure and exhaustion were very high, implying that any effects would need time to unfold. Therefore, we chose 6 months given that it is the most common time lag for longitudinal studies in organizational psychology (44).

Fourth, longitudinal studies inevitably suffer from non-response and attrition. The smaller database may bias the results and limit its generalizability. At the first occasion, we surveyed 3,420 students, at the second occasion 866 and at the last occasion 392. However, in order to examine whether the willingness to repeat was influenced by study variables, we compared participants who took part only at the first occasion with those who participated at T2 and T3 using *t*-tests. We did not find any significant differences between the participants, neither in the study variables nor in demographics (age, gender, workload).

Finally, our study was carried out at only one German university. It was not designed to examine differences between students from different universities and cultures, which would have required larger sample sizes. However, since the study conditions in Europe have been standardized due to the Bologna process, our results should also be relevant for other universities.

## Conclusion and Future Research

To our knowledge, our study is among the first that examined the relationship between time pressure and health-related loss of productivity mediated by exhaustion. There is a need for studies on health-related and non-health related causes of health-related productivity loss since these antecedents remain poorly understood and have rarely been investigated in university students. Our study further confirms SD-R's health impairment process longitudinally. The SD-R framework serves as an excellent theoretical basis to assess pathogenic effects of the study context on students' health and well-being. Specifically, we now know that time pressure constitutes one of the major demands at universities, leading to student burnout and impaired health in the long run. Therefore, we propose to implement interventions that address the pathogenic process in three dimensions:

First, study demands and especially time pressure due to an unequal distribution of workload need to be revisited (45, 46). Manageable workload has a positive effect on students' motivation and interest (47). However, there are almost no interventions that explicitly address time pressure for students by modifying study programs or structural settings at university. This is a large research gap that needs to be closed. We strongly advocate for an improvement of interventions on time pressure to prevent the negative consequences on students' health and well-being. Future research needs to focus on the conception and implementation of these interventions.

Second, students' time management should be ameliorated via time management training that helps students deal with time pressure. Whereas the first approach is directed toward the magnitude of demands, this approach intends to strengthen students' coping skills when dealing with time pressure. Time management training as an intervention has already been installed in educational settings (48), but is still not implemented as a regular offer for college students. Time management training is a promising tool to decrease perceived stress and increase perceived time control in university students (49).

Third, students' personal and interpersonal resources should be reinforced. Interpersonal resources such as supervisor support play a crucial role for preventing burnout (50). For university students, teacher support may be even more important than social support by for example, friends (51). Personal resources such as resilience also play a crucial role in students' health and well-being. Resilience training has positive effects on physicians (52), especially when combined with other intervention elements (53). So far, however, studies on the effects of resilience trainings at university have focused mainly on medical students (54, 55). Evidence for the general student population are missing.

Further research is needed to evaluate these approaches. In our view, interventions should address both, behavioral and structural changes in university (students). Since time pressure serves as the major study demand, we also propose regularly monitoring time pressure in students to prevent health-related productivity losses.

## Data Availability Statement

The original contributions presented in the study are included in the article/Supplementary Material, further inquiries can be directed to the corresponding author/s.

## Ethics Statement

The studies involving human participants were reviewed and approved by Ethics committee Freie Universität Berlin; FB Erziehungswissenschaft Psychologie. The patients/participants provided their written informed consent to participate in this study.

## Author Contributions

BG, TL, and CW: conceptualization, methodology, investigation, writing—review and editing, and project administration. BG and TL: validation, data curation, writing—original draft preparation, and visualization. BG: formal analysis and supervision. All authors: contributed to the article and approved the submitted version.

## Conflict of Interest

The authors declare that the research was conducted in the absence of any commercial or financial relationships that could be construed as a potential conflict of interest.
