# Supplementary material for: Time Pressure and Health-Related Loss of Productivity in University Students: The Mediating Role of Exhaustion
Source: Front Public Health. 2021 Apr 27;9:653440. doi: 10.3389/fpubh.2021.653440 (PMC8112546; doi:10.3389/fpubh.2021.653440)

Table. The items included in the student survey.

No.	Construct	Variable	Wording
1	Time Pressure	TP1	I have enough time to prepare and process the classes I attend.
2		TP2	I do not have enough time to complete study-related tasks with the appropriate care.
3		TP3	I feel overwhelmed by all the work my studies require.
4	Exhaustion	EX1	I feel emotionally drained by my studies.
5		EX2	I feel tired when I get up in the morning and I have to face another day at the university.
6		EX3	Studying or attending a class is really a strain for me.
7	Health-related Loss of Productivity	HLP1	Because of my acute health problems, the demands of my studies were much harder to handle.
8		HLP2	I was not able to focus on achieving my study goals, due to my acute health problems.
9		HLP3	My acute health problems distracted me from taking pleasure in my studies.
10		HLP4	I felt hopeless about finishing certain tasks, due to my acute health problems.
11		HLP5	I did not feel energetic enough to complete all my study-related tasks, due to my acute health problems.

Note. TP = Time Pressure; EX = exhaustion (MBI); HLP = Health-related Loss of Productivity; T1 = Time 1; T2 = Time 2; T3 = Time 3.

Table. Correlations of the study items.

			1	2	3	4	5	6	7	8	9	10	11	12	13	14	15	16	17	18	19	20	21	22	23	24	25	26	27	28	29	30	31	32
1	TP1	T1																																
2	TP2	T1	.65																															
3	TP3	T1	.59	.66																														
4	HLP1	T1	.28	.24	.36																													
5	HLP3	T1	.24	.23	.35	.72																												
6	HLP4	T1	.26	.23	.35	.76	.67																											
7	HLP2	T1	.25	.26	.35	.79	.75	.74																										
8	HLP5	T1	.27	.20	.34	.77	.73	.70	.79																									
9	EX1	T1	.42	.45	.62	.41	.42	.43	.39	.38																								
10	EX2	T1	.30	.30	.35	.30	.33	.32	.32	.34	.57																							
11	EX3	T1	.29	.35	.45	.40	.41	.44	.40	.42	.66	.57																						
12	TP1	T2	.59	.53	.50	.18	.19	.18	.18	.20	.36	.24	.24																					
13	TP2	T2	.47	.57	.56	.24	.22	.28	.27	.23	.43	.28	.37	.66																				
14	TP3	T2	.45	.48	.62	.34	.29	.35	.33	.32	.53	.35	.37	.60	.67																			
15	HLP1	T2	.10	.14	.24	.54	.46	.48	.48	.51	.30	.31	.39	.15	.25	.25																		
16	HLP3	T2	.12	.13	.26	.48	.47	.45	.49	.47	.36	.38	.39	.17	.24	.28	.80																	
17	HLP4	T2	.07	.16	.21	.51	.47	.53	.51	.50	.34	.27	.40	.14	.25	.27	.80	.74																
18	HLP2	T2	.10	.14	.23	.53	.47	.51	.51	.51	.30	.34	.39	.16	.26	.26	.85	.82	.78															
19	HLP5	T2	.08	.14	.22	.49	.43	.45	.44	.47	.31	.35	.38	.18	.24	.27	.85	.82	.74	.84														
20	EX1	T2	.38	.41	.56	.37	.38	.44	.41	.36	.67	.50	.56	.47	.58	.65	.34	.39	.37	.34	.37													
21	EX2	T2	.25	.31	.36	.28	.33	.36	.35	.35	.47	.62	.52	.35	.40	.41	.36	.41	.37	.36	.40	.67												
22	EX3	T2	.28	.38	.43	.36	.36	.41	.39	.38	.53	.50	.64	.36	.49	.46	.45	.47	.44	.46	.46	.68	.69											
23	TP1	T3	.49	.48	.51	.17	.23	.21	.22	.17	.34	.19	.24	.61	.56	.49	.15	.16	.14	.12	.14	.44	.29	.30										
24	TP2	T3	.41	.49	.46	.20	.21	.26	.24	.21	.36	.29	.32	.49	.61	.51	.24	.23	.20	.23	.23	.44	.33	.36	.63									
25	TP3	T3	.43	.50	.60	.24	.28	.27	.28	.24	.51	.33	.39	.49	.54	.64	.27	.31	.27	.26	.28	.58	.41	.44	.57	.68								
26	HLP1	T3	.13	.22	.23	.49	.43	.41	.44	.46	.32	.31	.42	.13	.17	.20	.49	.48	.44	.46	.49	.34	.33	.40	.22	.24	.32							
27	HLP3	T3	.14	.22	.28	.47	.46	.43	.46	.48	.38	.33	.36	.18	.21	.23	.43	.49	.43	.44	.47	.36	.31	.40	.22	.25	.34	.80						
28	HLP4	T3	.18	.26	.28	.48	.44	.46	.48	.48	.34	.31	.41	.17	.23	.18	.48	.51	.47	.49	.49	.36	.32	.42	.23	.30	.34	.82	.81					

29	HLP2	T3	.15	.27	.27	.50	.46	.46	.48	.48	.32	.29	.37	.14	.22	.21	.48	.47	.48	.47	.47	.33	.31	.38	.25	.26	.30	.84	.80	.84				
30	HLP5	T3	.16	.24	.27	.48	.47	.45	.48	.49	.34	.33	.39	.16	.23	.25	.45	.46	.44	.47	.46	.36	.36	.41	.26	.29	.36	.83	.80	.80	.85			
31	EX1	T3	.36	.45	.55	.32	.34	.35	.36	.32	.63	.47	.53	.41	.51	.53	.33	.38	.34	.31	.35	.69	.56	.56	.47	.52	.68	.39	.44	.43	.40	.43		
32	EX2	T3	.25	.34	.37	.33	.35	.40	.38	.39	.48	.56	.52	.28	.36	.37	.37	.39	.38	.36	.38	.54	.61	.53	.35	.41	.47	.41	.44	.44	.44	.46	.71	
33	EX3	T3	.27	.36	.46	.35	.38	.37	.35	.37	.53	.46	.58	.33	.43	.42	.40	.39	.38	.39	.38	.55	.50	.62	.39	.44	.52	.44	.46	.48	.45	.48	.71	.70

Note. TP = Time Pressure; EX = exhaustion (MBI); HLP = Health-related Loss of Productivity; T1 = Time 1; T2 = Time 2; T3 = Time 3.

Figure. The reversed model (M2).

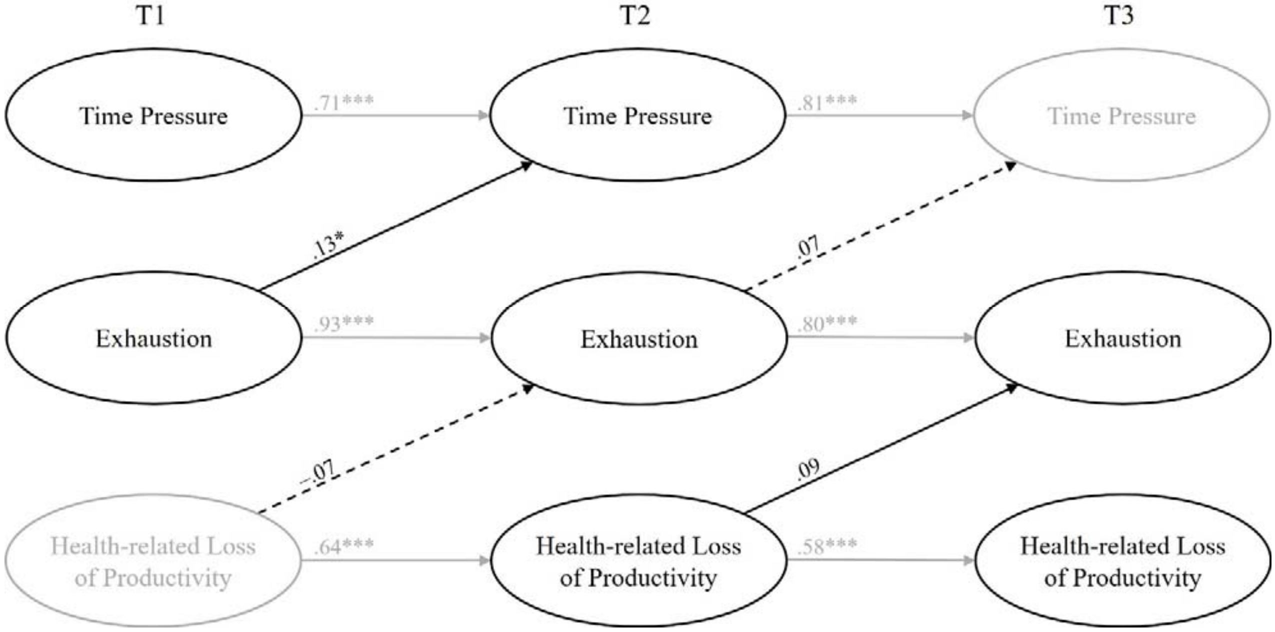

Figure. The reciprocal model (M3).

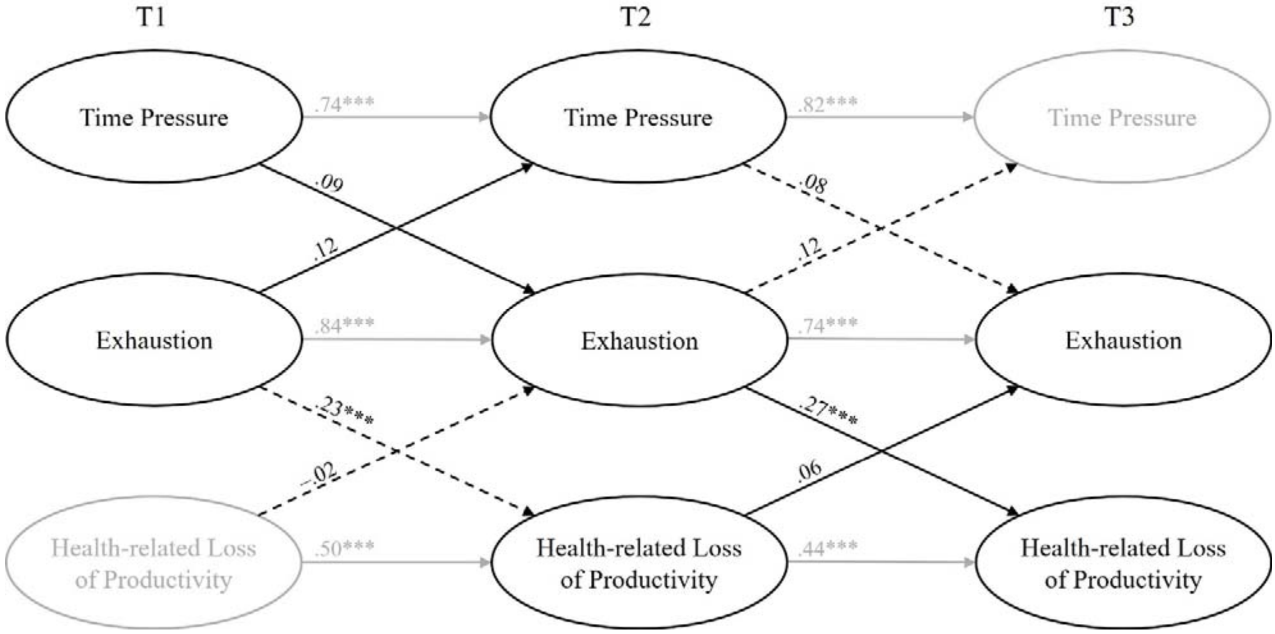

Supplement: Supplementary file 1 [file Data_Sheet_1.PDF]
